# Level, Source, and Spatial Distribution of Potentially Toxic Elements in Agricultural Soil of Typical Mining Areas in Xiangjiang River Basin, Hunan Province

**DOI:** 10.3390/ijerph17165793

**Published:** 2020-08-10

**Authors:** Yang Yu, Haijiang Luo, Lihuan He, Wenqing Liu, Renji Xu, Linlin Zhang, Guihua Dong, Yeyao Wang, Guoping Wu, Fusheng Wei

**Affiliations:** 1 Anhui Institute of Optics and Fine Mechanics, Hefei Institutes of Physical Science, Chinese Academy of Sciences, Hefei 230026, China; yuyang_eco@cnemc.cn (Y.Y.); wqliu@aiofm.ac.cn (W.L.); 2 University of Science and Technology of China, Hefei 230026, China; 3 State Environmental Protection, Key Laboratory of Quality Control in Environmental Monitoring, China National Environmental Monitoring Center, Beijing 100012, China; luohj@cnemc.cn (H.L.); helh@cnemc.cn (L.H.); xurj@cnemc.cn (R.X.); zhangll@cnemc.cn (L.Z.); donggh@cnemc.cn (G.D.); wangyy@cnemc.cn (Y.W.); spchina@263.net (G.W.)

**Keywords:** soil pollution, source identification, multivariate analysis, risk evaluation

## Abstract

The concentrations, chemical availability, distribution, and sources of potentially toxic elements (PTEs) in the soil of Xiangjiang Basin in Hunan Province, China were investigated at 85 sites. The highest mean concentrations of Cd, Cu, Zn, As, and Pb were observed in Hengyang, whereas those for Mn, Co, and Hg were observed in Changde. The pollution index values followed the order: Cd > Hg > Cu > Zn > As > Pb; the mean geo-accumulation index values were in the order: Cd > Hg > Pb > Cu > Zn > As > Co > Mn. Cd was associated with moderate contaminated level, Hg and Pb were associated with moderate contaminated to uncontaminated level, and Cu, Zn, As, Co, and Mn were associated with uncontaminated level of pollution. Furthermore, 64.5% of Cd was water-soluble and exhibited exchangeable fractions; its chemical availability posed a risk to the ecosystem. Spatial analysis, principal component analysis, and a positive matrix factorization model were used to assess the PTE sources. Four principal components contributed to 88.8% of the 8 PTEs concentrations. Mining, smelting, industrial, and agricultural activities, alongside sewage irrigation, the use of agrochemicals, and vehicular emissions are the possible anthropogenic sources that pollute agricultural products and threaten human health in the Xiangjiang Basin.

## 1. Introduction

Potentially toxic elements (PTEs) in soil refer to substances that are introduced into the soil as a result of human activities and can alter the quality and function of the soil. This leads to soil degradation and damages the basic soil structure, potentially causing harm to human health and the environment [1,2,3]. China is one of the largest global producers and consumers of metals and metalloids. A total of 171 mineral varieties are found in the Chinese territory, which account for 12% of the world’s mineral resources and contribute to the country’s socio-economic development [4]. According to a national soil contamination survey covering the period 2005–2013 [5], soil pollution in China is a major problem, as 16.1% of the analyzed soil samples exceeded the national environmental quality standards.

Hunan Province in China is renowned for its nonferrous metals; hence, the supply and manufacturing chain related to nonferrous metals is well developed. Despite a high economic production, nonferrous metal manufacturing causes environmental damage, such as a significant amount of PTEs pollution in the soil [6,7]. Out of the 30 national heavy metal pollution prevention and key control areas, 11 are located in Hunan Province. These areas are mainly distributed in nonferrous metal mineral areas and the Xiangjiang River Basin, including the Changzhutan area, the Hengyang Shuikou Mountain, and Changshaoyue [8].

Hunan is also a renowned rice production area. Rice production reaches 2.32 × 10^7^ t/y, which is the largest in China, and accounts for 12.7% of the national total [9]. However, soil pollution in Hunan exceeds the environmental quality standards for PTEs, which severely affects agricultural development [10]. Therefore, the examination of soil pollution and land use in Hunan Province is crucial to ensure healthy agricultural production. Previous studies mostly focused on PTE remediation, removal from irrigation water, absorption and accumulation mechanisms in agricultural products, and risk assessments [11,12,13,14,15]. Reports on PTEs in the soils of regions featuring multiple land-use patterns are scarce. To better understand the current state of PTE pollution in Hunan soil, this study focused on investigating a region with developed industry, dense population and large-scale grain production. The main objective of the study was to evaluate the concentration, spatial distribution, and potential sources of PTEs in agricultural soil in typical polluted areas in Hunan. The findings provide information on PTE pollution control in soil, supporting the formulation of environmental protection measures in the Xiangjiang Basin.

## 2. Materials and Methods

### 2.1. Soil Sampling and Preparation

**Figure 1:**
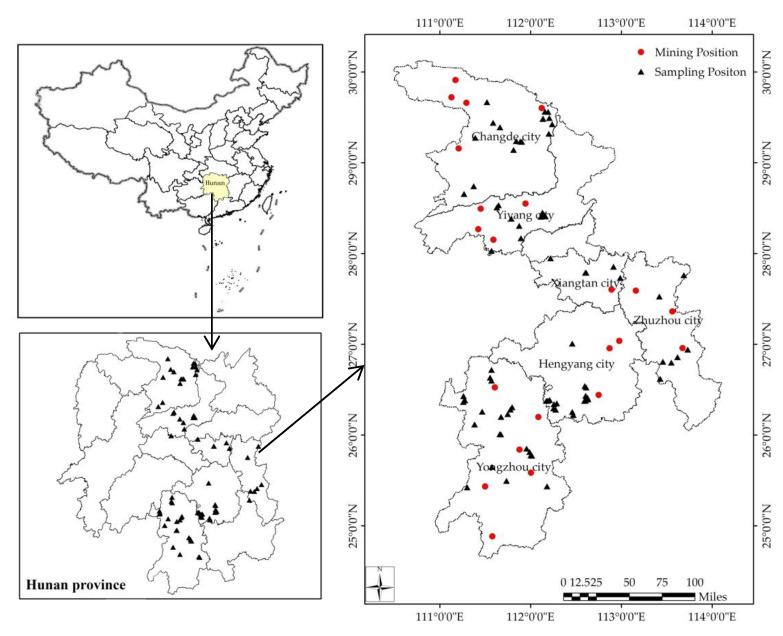
Maps showing the sampling and mining locations.

This study was conducted in Hunan Province, China, and it focused on characterizing and evaluating soils from six cities along the Xiangjiang River. Based on the historical data on the concentration of PTEs in soil in Hunan Province [16], the 85 sampling locations and mining areas investigated in this study [17] are shown in Figure 1.

The soil samples were collected from the upper horizon (0–20 cm) of soils across a 50 × 50 m area on each sampling site. In total, 5 samples were taken at each location using the plum blossom method, in which soils are taken from the four corners and the center of an area and then blended. Nearly 2.5 kg of sampled soils were thoroughly mixed prior to testing [18]. In the laboratory, the soil was first spread out on a piece of Kraft paper (80 × 110 cm) in an air-drying room where the temperature was lower than 35 °C, forming a 2 cm thick layer. After removing plant leaves, crushed stones, and other impurities, the samples were left to dry naturally. Then, they were ground and sieved through a 0.15 mm sieve prior to being submitted to further analyses.

### 2.2. Sample Analysis

#### 2.2.1. Potentially Toxic Elements Content

For each sample, 0.20 g of soil was digested with a mixture of 5 mL HNO_3_, 1 mL HClO, and 1 mL HF and processed following the methods specified by the Chinese national standard for measuring PTEs in soil [19]. The concentrations of Cd, Mn, Cu, Zn, Co, As, and Hg were analyzed by inductively coupled plasma–mass spectrometry (ICP-MS, Agilent, 7700x, Santa Clara, CA, USA). The total Pb in soil was analyzed via X-ray fluorescence spectrometry [20].

#### 2.2.2. Chemical Availability

Five grams of prepared soil were placed in a 100 mL cone bottle, and 25 mL diethylenetriamine pentaacetic acid (DTPA) was added with a pipette. The suspensions were shaken at 200 rpm for 2 h at 25 °C, centrifuged at 8000 rpm for 10 min, and filtered through 0.45 µm filter paper. The concentrations of Cd, Mn, Cu, Zn, Co, Hg, As, and Pb were analyzed via ICP-MS (Agilent 7500a, Santa Clara, CA, USA) following the standard procedures (GB/T 23739-2009) [21].

#### 2.2.3. Soil pH

The air-dried soil samples were filtered through a 0.15 mm sieve, and 10 g of sample was added into a 25 mL beaker with 10 mL of distilled water. The mixture was kept still for 30 min, and the pH of the suspension was measured using a pH meter (METTLER, S220, Zurich, Switzerland).

### 2.3. Data Analysis

The SPSS software (IBM SPSS Statistics for Windows, Version 20.0. Armonk, NY: IBM Corp, Amonk, NY, USA) was used to perform the one-way analysis of variance. The spatial distribution of PTEs was mapped using ArcGIS 10.0 (ESRI, Redlands, CA, USA). Spearman’s correlation was conducted to examine the relationships among the potentially toxic elements. A level of significance of 0.05 or lower was chosen. Principal component analysis (PCA) and positive matrix factorization (PMF) modeling (USEPA PMF 5.0) were performed to assess the sources of the PETs.

### 2.4. Evaluation Methods

To assess the degree of PTEs contamination, the pollution index (PI) for each PTE and the overall PTEs pollution status of the soil were assessed through the Nemerow integrated pollution index (NIPI) [22]:

(1) $$\mathrm{PI}=\frac{C_{i}}{S_{i}}$$

where C_i_ is the measured concentration of each PTE (Cd, Cu, Hg, Pb, Zn and As) in this study, and S_i_ is the screening value for the pollution risk of the respective PTEs in soil. The PTEs pollution status of the paddy soil was evaluated with respect to the screening value for soil pollution risk in agricultural land specified in the “Soil Environmental Quality Agricultural Land Pollution Risk Control Standard (GB 15618-2018)” [23] (Table 1). The PI of each PTE was classified as non-pollution (PI < 1), low level of pollution (1 ≤ PI < 2), moderate level of pollution (2 ≤ PI < 3), high level of pollution (3 ≤ PI < 5), and very high level of pollution (PI > 5). The NIPI of each PTE in each sample was calculated as follows:

(2) $$\mathrm{NIPI}=\frac{{\mathrm{PI}}_{\mathrm{iave}}^{2}+{\mathrm{PI}}_{\mathrm{imax}}^{2}}{2}$$

where ${\mathrm{PI}}_{\mathrm{imax}}$ is the maximum PI value for each PTE, and ${\mathrm{PI}}_{\mathrm{iave}}$ is the respective mean PI value. The NIPI was classified as non-pollution (NIPI ≤ 0.7), warning line of pollution (0.7 < NIPI ≤ 1), low level of pollution (1 < NIPI ≤ 2), moderate level of pollution (2 < NIPI ≤ 3), and high level of pollution (NIPI > 3) [22].

The geo-accumulation index ($I_{\mathrm{geo}}$) quantifies the impact of an anthropogenic factor with respect to an environmental geochemistry background [24]. It is calculated as follows:

(3) $$I_{\mathrm{geo}}=\log_{2}^{\left[\frac{C_{i}}{1.5B_{i}}\right]}$$

where $C_{i}$ is the concentration of the PTE (Cd, Cu, Hg, Pb, Zn, Mn, Co, and As) in the soil sample, and $B_{i}$ is the geochemical background concentration of the PTE (n) (Table 1). A coefficient of 1.5 is introduced as a background matrix correction factor owing to the lithological variability of the soils [25]. $I_{\mathrm{geo}}$ is classified into seven grades or classes [26]: Class 0 (uncontaminated), $I_{\mathrm{geo}}$ ≤ 0; Class 1 (from uncontaminated to moderately contaminated), 0 < $I_{\mathrm{geo}}$ ≤ 1; Class 2 (moderately contaminated), 1 < $I_{\mathrm{geo}}$ ≤ 2; Class 3 (from moderately to strongly contaminated), 2 < $I_{\mathrm{geo}}$ ≤ 3; Class 4 (strongly contaminated), 3 < $I_{\mathrm{geo}}$ ≤ 4; Class 5 (from strongly to extremely contaminated), 4 < $I_{\mathrm{geo}}$ ≤ 5; Class 6 (extremely contaminated), 5 < $I_{\mathrm{geo}}$. Class 6 is an open class and comprises all values of the index higher than Class 5. The elemental concentrations in Class 6 may be 100-fold greater than the geochemical background value.

## 3. Results

### 3.1. Descriptive Statistics

The PTE concentrations in the soil samples are listed in Table 1. The mean concentrations of Mn, Zn, Pb, Cu, As, Co, Cd, and Hg were 431.2, 133.3, 49.15, 45.3, 17.01, 13.29, 1.31, and 0.81 mg/kg, respectively. Compared to the local background values, the mean concentrations of Cd, Hg, Cu, Pb, Zn, and As exceeded the background levels by 1.13–10.08 times, with percentages of 907.7%, 450.0%, 65.9%, 65.5%, 41.2%, and 13.4%, respectively. In contrast, the mean concentrations of Mn and Co were lower than the background values at most sites.

### 3.2. Spatial Distribution

**Figure 2:**
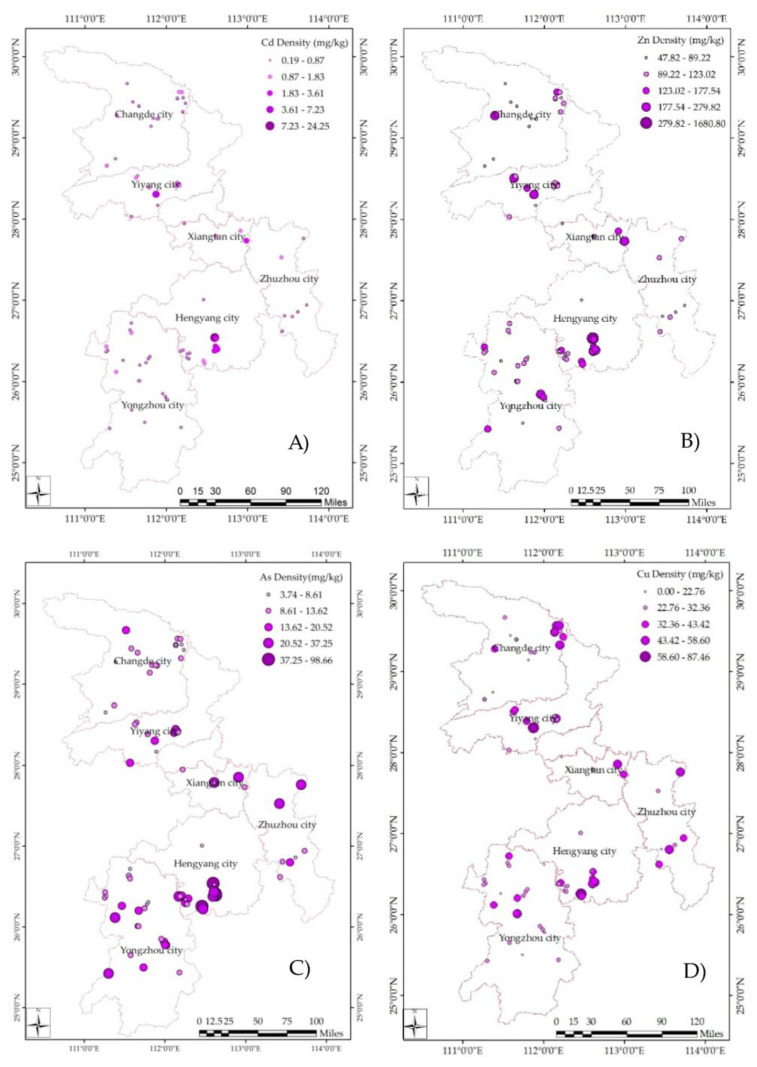

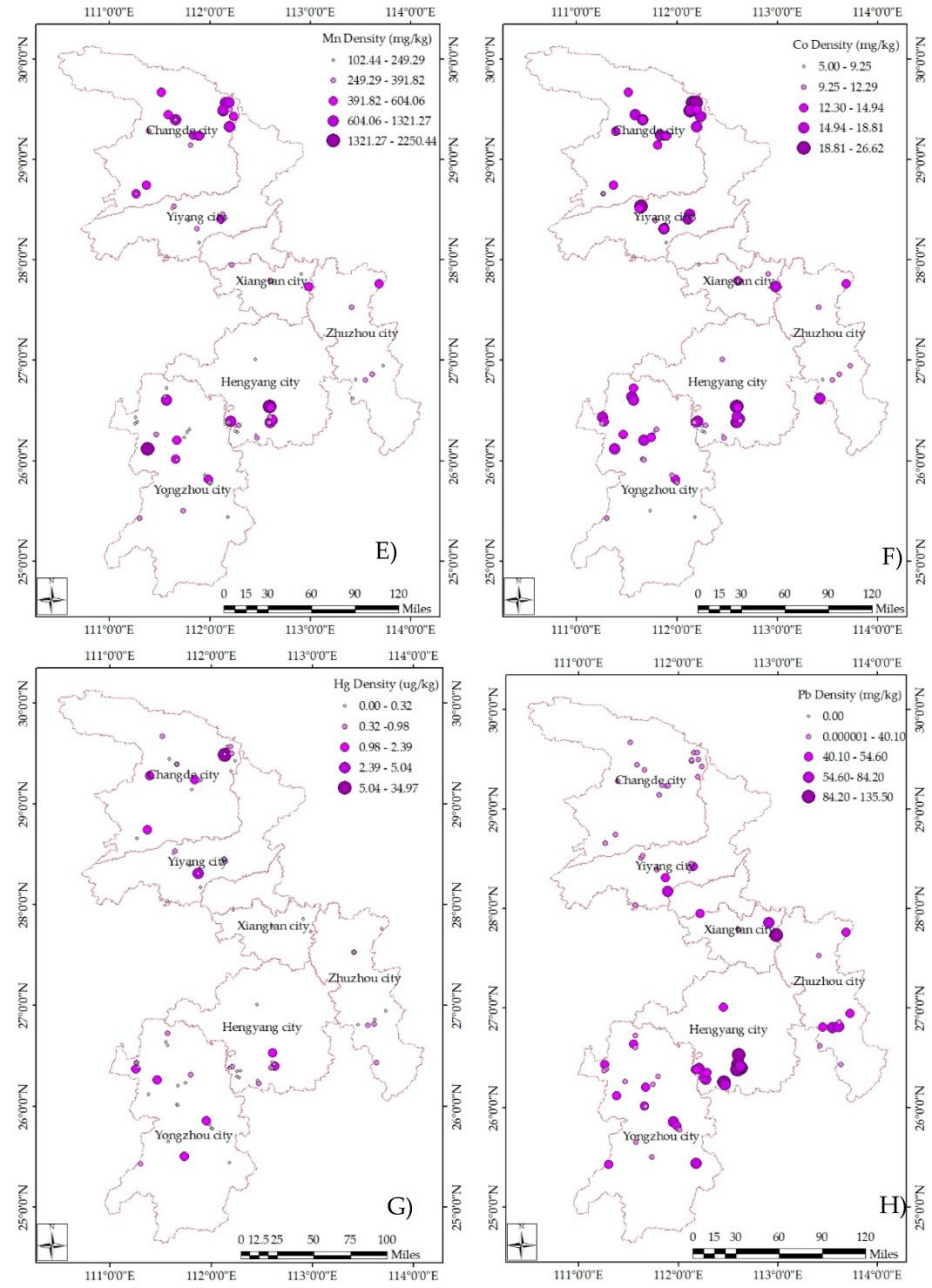
PTEs spatial distribution in the soil of six cities in Hunan based on 85 samples: (**A**) Cd, (**B**) Zn, (**C**) As, (**D**) Cu, (**E**) Mn, (**F**) Co, (**G**) Hg, and (**H**) Pb.

The spatial distribution of the PTEs in soil is shown in Figure 2. The concentrations decrease with increasing distance from each peak. The highest mean Cd, Cu, Zn, As, and Pb concentrations were 3.12, 97.19, 235.08, 34.35, and 85.96 mg/kg, respectively, and were observed in Hengyang. The highest mean Mn, Co, and Hg concentrations were 534.88, 15.13, and 1.87 mg/kg, respectively, and were observed in Changde (Table 1).

The spatial distribution of Cd, As, and Hg were similar. High concentrations of these PTEs were found in four cities: Changde, Yiyang, Xiangtan, and Hengyang. In the northeast of Changde, a circular region of high Mn concentration extended from locations where large-scale Mn mining and steel works were distributed. Co and Zn had similar distributions to Mn. The highest concentrations of Pb were reported south of Hengyang, where a large Pb/Zn mine was located. Cu exhibited a similar distribution to Pb.

### 3.3. Chemical Availability

In this study, chemical availability refers to the number of desorbed elements (from the total concentration) that are readily available for plant uptake [27]. The chemical availability of PTEs in soil is related to their total concentrations and depends on environmental conditions [28]. The bioavailable fraction of a PTE is particularly important when considering the contamination of agricultural production, since these contaminants can easily be absorbed by plants. Among the PTEs assessed in this study, Cd presented the highest mean chemical availability percentage (bioavailable concentration to the total concentration) of 64.54%, followed by Mn and Pb. Similar results were obtained by Sun et al. [29]. In contrast, the chemical availability percentages of Cu, Zn, As, and Hg were relatively low (less than 4%) (Table 2).

### 3.4. PTEs Pollution

Based on the soil quality data of the study area, a quantitative analysis of PTE pollution in the soil was conducted using the PI and NIPI methods. The mean concentrations of Mn and Co were lower than their background concentrations, and currently, there are no screening value standards for these elements in GB15618-2018 [23]. Therefore, the concentrations of Mn and Co were not included in the PI and NIPI evaluations. The PI ranges for Pb, Zn, and Cu in the sampled soil were 0.11–1.07, 0.24–1.40, and 0.20–1.55, respectively, indicating that the soil samples have zero or low levels of pollution. The PI value for As in the sampled soil was 0.09–2.82, indicating non-pollution to moderate levels of pollution. The PI values for Cd and Hg were 0.48–40.42 and 0.01–5.04, respectively, indicating non-pollution to very high level pollution. The mean PI values ranked in descending order are as follows: Cd (2.58) > Hg (1.05) > Cu (0.52) > Zn (0.51) > As (0.47) > Pb (0.41) (Table 3). The overall NIPI was 28.59, which indicates a high level of pollution.

The I_geo_ values were used as a reference to estimate the extent of PTE pollution (Table 4). The mean I_geo_ values ranked in descending order were Cd (1.97) > Hg (0.81) > Pb (0.02) > Cu (–0.31) > Zn (–0.35) > As (–0.72) > Co (–0.78) > Mn (–1.04). Therefore, the I_geo_ results indicate uncontaminated to moderately contaminated levels of PTE pollution in the study area. Cd was associated with a moderate contaminated levle (1< I_geo_ ≤2), Hg and Pb were associated with uncontaminated to moderately contaminated levle (0 < I_geo_ ≤ 1), and Cu, Zn, As, Co, and Mn were associated with uncontaminated levle (I_geo_ ≤0).

## 4. Discussion

### 4.1. PTEs Pollution

The PI results are listed in Table 3. The Pb, Zn, and Cu concentrations in the sampled soil exceeded the GB15618-2018 standards in some locations [23]; however, the overall concentrations were relatively low. The single-factor PIs in the areas that exceeded the standards exhibited low levels of Pb, Zn, and Cu pollution. The concentrations of Cd and Hg represented high levels of pollution, with pollution rates of 70.59% and 36.21%, respectively (Table 3). Previous studies have shown that the concentration of Cd in farmland soil in China is 0.20 mg/kg in the east, 0.21 mg/kg in the north, 0.25 mg/kg in the northeast, 0.33 mg/kg in the west, and 0.51 mg/kg in the south. Hunan Province falls under the southern region with respect to this sub-classification [30]. This indicates that the 1.31 mg/kg mean concentration of Cd in the soil of the sampled area is higher compared to other parts of China.

The overall NIPI was 28.59, which indicated a high level of pollution; Cd, Hg, and Cu were the main contributing PTEs. The I_geo_ values showed that Cd, Hg, and Pb were the main contributing factors, with contamination rates of 97.6%, 61.0%, and 35.8%, respectively, and the average value of Cd indicated a moderate level of pollution. The obvious enrichment of these PTEs in soil should be noted.

### 4.2. Spatial Distribution

It is known that Cd, As, Cu, and Zn exhibit similar chemical behaviors, and their environmental concentrations are highly influenced by human activities, such as mining. The Shuikoushan mine (Songbai Town, Hengyang City, China) is the largest and oldest mine in Hunan Province, covering the area where the highest concentrations of Cd, Pb, and Zn have been recorded. It produced 210,000 t of Pb and 540,000 t of Zn between 1869 and the 1900s [31]. The PTE contamination caused by mining and processing at this site has continuously polluted the local soil.

Pb pollution hotspots include the mining region in southern Hengyang and the densely populated residential areas of Xiangtan City, which are possibly strongly associated with anthropogenic inputs from vehicle emissions and Pb-based paints [32,33]. In addition, sewage from mining and sand washing is often used to irrigate farmlands, and smelting slag is stacked near farmlands, which then flows into the fields through rainwater leaching and infiltration. Many factors can lead to large amounts of PTEs entering the ground, resulting in sharp increases in PTE concentrations in the soil.

The variations in Mn and Co concentrations in the sampled soil were similar. Regions with high concentrations of these elements were located on the eastern edge of Changde, in the center of Yongzhou, and on the eastern edge of Zhuzhou. Although the average concentrations of Mn and Co were lower than their respective background values, according to the monitoring results from 85 sites, the concentrations were much higher in these areas. In China, Mn ores are distributed unevenly and are concentrated in the southwestern provinces [34]. By the end of 2015, the total Mn ore reserves in Hunan Province were estimated at approximately 172 Mt. Yongzhou City has the largest reserves, at approximately 54.09 Mt, accounting for 31.41% of the Mn ore reserves in Hunan [35].

The mean concentration of Hg was higher than its background value and varied widely among different sites. The concentration was high in the center of Yiyang, in the south of Hengyang, and at the western edge of Changde. A previous study showed that Yiyang is located in the largest Sb mining area in the world [36]. The Sb mining and smelting activities lead to the introduction of Sb and other PTEs, such as As and Hg, into the surface soil, which then pollutes the farmlands in the mining area and endangers human health. Furthermore, the Hg content of the soil in smelting areas is higher than in mining areas, which in turn is higher than in tailing areas. Nonetheless, the contents in all the above areas are much higher compared to the control areas and the soil background value in Hunan Province [37]. The mean concentration observed in the previous study was 0.50 mg/kg, which is similar to the value obtained in this study.

### 4.3. Source Apportionment

The Spearman’s rank correlation coefficients between the assessed PTEs are summarized in Table 5. Positive correlations were observed between Zn, Cd, Cu, and As, suggesting related origins of these elements. However, the correlations between these PTEs are complex and influenced by various factors. Therefore, no definitive conclusions could be drawn from the Spearman’s rank correlations. To further examine the relationship between the PTEs and their sources, PCA and a PMF model were used. The results of the PCA were confirmed by a KMO (Kaiser Meyer Olkin) test and the sampling efficiency was verified. Using PCA, four principal components were extracted that cumulatively accounted for 88.8% of the total variance in the PTE concentrations (Table 6). According to the rotated component matrix, the main component is a combination of Zn, Cd, Cu, and As, which represents 41.36% of the total variance, whereas Mn and Co collectively account for 21.14% of the variance (component two). The third component is Pb and the fourth is Hg. It was assumed that variables with similar loadings present a stronger mutual correlation [38].

According to satellite remote sensing observations by Liu et al. [39], the mining area of Hunan Province accounts for 0.27% of the total land area of the province. The mining areas in Hengyang, Yongzhou, Zhuzhou, Changde, Yiyang, and Xiangtan account for 14.93%, 13.22%, 6.09%, 4.6%, 4.42%, and 3.07% of the total mining areas of the provinces, respectively. Hengyang and Yongzhou are ranked first and third, with higher mining area proportions than the provincial mean value. The results show that all six cities feature mining activities, leading to possibly varying levels of soil pollution [39].

Component 1 predominantly consists of Zn, Cd, Cu, and As. According to the results of a survey conducted at 147 agricultural sites in Hunan Province by other scholars, the concentration of Cd in agricultural land is 7.69 mg/kg, which is significantly higher than the average in the province, indicating that agricultural activities are also a major contributing factor to soil pollution [30]. Previous research has indicated that the main sources of PTEs in the soil of the study area include pollutant discharge from businesses, pesticides, chemical fertilizers, and automobile exhausts related to agricultural activities [40]. For example, Cd is usually a marker element for agronomic practices such as the use of chemical fertilizers, including phosphate fertilizers and livestock manure [41]. In addition, Zn and its compounds are widely used in agricultural fertilizers, and cattle slurry can contain significant concentrations of Zn and Cu, because these elements are fed to cattle as trace PTEs supplements.

Component 2 is dominated by Mn and Co. As reported in previous studies, the Mn mineral processing output in Yongzhou is the highest in Hunan Province. By the end of 2012, 65 types of 168 minerals were extracted or processed in Yongzhou City. Hunan Province has the largest reserves of Mn, Rb, Li, and rare earth elements in China. Cobaltite and pyroxene are the main Co minerals in nature, and the production of Co is often associated with the mining of Mn; there is a strong correlation between Mn and Co sources. Xu et al. [42] observed that the parent material and pedogenic processes lead to the accumulation of As in the soil.

Component 3 is dominated by Pb. It is generally believed that the concentrations of Pb and Zn should show the same trend due to the influence of Pb–Zn mining. However, it was found that Pb and Zn belonged to different components. Industrial activities are likely the main cause of Pb pollution. Moreover, Pb hotspots also include densely populated residential areas (city centers), which may be strongly associated with anthropogenic inputs such as vehicle emissions and Pb-based paints. Indeed, Pb is typically a marker for vehicle emissions [32].

Component 4 was dominated by Hg. An inventory of Hg emissions from anthropogenic activities in China in 1999 was compiled by Streets et al. [43] using official statistical data. They estimated that the total Hg emission in China was 536 ± 236 t. This value includes open biomass burning and excludes natural sources or the re-emission of previously deposited Hg. The authors also reported that approximately 38% of Hg emission in China arises from coal combustion, 45% arises from nonferrous metal smelting, and 17% arises from miscellaneous activities. The main Hg concentration hotspots in this study were observed in Hengyang, Yiyang, and Yongzhou, where large-scale PTEs mines and steel works are located. These activities release Hg-rich effluents into the surrounding environment. The toxic substances in the waste may transfer to reclaimed soil through leaching and weathering, potentially causing severe soil pollution [44].

### 4.4. Chemical Availability

According to Feng et al. [45], the concentrations of PTEs in plants are related to their uptake from the soil. In a study on the speciation and chemical availability of PTEs in soil, Guo [12] showed that Pb, Zn, and Cd migrated more easily than other PTEs and were therefore more readily available for utilization by plants; thus, they represented a greater ecological risk. The PI value for Cd was the highest in the current study, which may be related to wastewater used for irrigation and chemical fertilization [46]. The mobility and chemical availability of PTEs strongly depend on the soil properties and particularly on its pH. In this study, the mean pH was 6.38 (Table 2), suggesting that the soil in the study area was generally acidic. Previous studies have shown a significant negative correlation between the pH of the soil and the water-soluble and exchangeable fractions of Cd [15]. Based on this, the study area can be deemed conducive to the absorption of PTEs by agricultural products. Therefore, agricultural products planted in the area are more likely to be polluted, and more attention should be paid to the safety of edible crops.

## 5. Conclusions

This study provides original data concerning the levels, chemical availability, spatial distribution, and possible sources of PTEs in the soil of a representative region of the Xiangjiang Basin in Hunan Province. The highest mean concentrations of Cd, Cu, Zn, As, and Pb were observed in Hengyang. The highest mean concentrations of Mn, Co, and Hg were observed in Changde. The mean concentrations of Zn, Cu, Pb, As, Cd, and Hg in the sampled soil exceeded their background levels, whereas those of Mn and Co were lower than their background levels. According to the results of the single factor evaluation and accumulation index, Cd poses the highest risk to human health and the environment in this region.

The spatial distribution trends show positive correlations between Zn, Cd, Cu, and As, suggesting related origins of these elements. Furthermore, a strong correlation between PTE concentrations and former mining, agricultural, and industrial activities was found. Owing to various pollution sources and soil conditions, the local agricultural products of this region are more likely to be polluted than those produced in other regions of China. Furthermore, there is a need to pay closer attention to ensure the safety of these agricultural products. The findings of this study can be used to manage PTE pollution in soil in the study area. Consequently, they can also be used to aid the preparation of environmental protection measures in the Xiangjiang Basin in Hunan Province.

## Figures and Tables

**Table 1:** Descriptive statistics of potentially toxic elements (PTEs) concentrations in the surface and sub-surface soils (mg/kg).

Element	Concentration (mg/kg)	City							Background Value (mg/kg) [16]	Screening Value (mg/kg) [23]
		Yongzhou	Zhuzhou	Yi Yang	Changde	Heng Yang	Xiang Tan	Six Cities		
**Cd**	min	0.19	0.34	0.31	0.26	0.52	0.31	0.19	0.13	0.3–0.8
	max	1.40	0.95	7.23	1.60	24.25	2.16	24.25		
	Mean	0.67	0.66	1.99	0.59	3.12	0.88	1.31		
	SD	0.30	0.21	2.43	0.33	5.72	0.72	2.77		
	CV (%)	44.8	31.8	122.1	55.9	183.3	81.8	211.5		
Cu	min	17.47	22.77	21.03	15.9	20.54	17.11	15.90	27.3	50–100
	max	47.27	55.51	77.55	58.24	993.41	47.80	993.41		
	Mean	29.50	37.20	41.38	32.70	97.19	29.72	45.31		
	SD	7.20	11.39	18.83	13.54	238.50	11.65	104.92		
	CV (%)	24.4	30.6	45.5	41.4	245.4	39.2	231.6		
Zn	min	64.06	66.30	74.03	47.83	78.76	51.30	47.83	94.4	200–300
	max	202.18	120.53	249.34	279.83	1680.76	276.25	1680.8		
	Mean	107.03	91.96	142.09	95.01	235.08	124.92	133.28		
	SD	28.58	20.30	61.74	49.20	388.28	82.69	176.94		
	CV (%)	26.7	22.1	43.5	51.8	165.2	66.2	132.8		
As	min	4.57	8.41	3.75	4.08	5.63	6.43	3.75	15.0	40–25
	max	25.11	31.10	18.59	20.53	98.66	29.00	98.66		
	Mean	13.30	16.77	12.93	10.67	34.35	15.17	17.01		
	SD	4.90	8.74	4.21	3.78	29.60	8.91	15.95		
	CV (%)	36.8	52.1	32.6	35.4	86.2	58.7	93.8		
Mn	min	102.44	143.52	128.56	194.82	110.37	177.42	102.44	459	-
	max	2250.4	478.30	453.53	903.02	1971.15	528.45	2250.4		
	Mean	443.76	281.80	260.00	534.88	519.82	315.41	431.18		
	SD	577.43	118.02	99.20	216.21	490.54	121.22	398.88		
	CV (%)	130.1	41.9	38.2	40.4	94.4	38.4	92.5		
Co	min	5.00	8.27	5.53	6.05	7.41	7.18	5.00	14.6	-
	max	18.81	14.44	19.87	21.62	26.63	15.16	26.63		
	Mean	12.64	11.06	13.38	15.13	13.15	11.54	13.29		
	SD	3.56	3.11	3.72	3.66	4.65	2.84	3.82		
	CV (%)	28.2	28.1	27.8	24.2	35.4	24.6	28.7		
Pb	min	0	37.8	28.7	26.4	0	34.5	0	29.7	80–240
	max	73.0	65.0	58.8	37.2	288.5	107.1	288.50		
	Mean	41.91	46.92	39.58	32.05	85.96	58.38	49.15		
	SD	13.21	10.42	7.66	2.68	64.78	26.99	35.12		
	CV (%)	31.5	22.2	19.4	8.4	75.4	46.2	71.5		
Hg	min	0.0	0.0	0.0	0.0	0.0	0.02	0.0	0.12	0.5–1.0
	max	1.49	2.06	0.17	34.97	5.04	0.32	34.97		
	Mean	0.33	0.72	0.04	1.87	1.07	0.19	0.81		
	SD	0.43	0.80	0.07	7.80	1.25	0.10	3.84		
	CV (%)	130.3	111.1	175.0	417.1	116.8	52.6	474.1		

**Table 2:** Chemical availability of PTEs in soil (%).

Concentration (mg/kg)	Mn	Co	Cu	Zn	As	Cd	Pb	Hg
N	85	85	85	85	85	85	85	59
Min	8.16	4.15	0.05	0.20	0.07	14.12	0.00	0.01
Max	87.45	29.16	19.96	10.89	9.61	100.00	38.51	3.07
Mean	33.205	12.01	3.78	3.26	1.74	64.54	10.48	0.08
SD	17.21	5.62	4.19	1.94	1.87	22.31	8.97	0.34
CV	296.13	31.63	17.57	3.76	3.494	497.68	80.41	0.12

SD: standard deviation; CV: coefficient of variation.

**Table 3:** Pollution index (PI) and Nemerow integrated pollution index (NIPI) values.

Element		Pi						NIPI
		Cd	Pb	Zn	Hg	Cu	As	
Max (mg/kg)		40.42	1.07	1.40	5.04	1.55	2.82	
Min (mg/kg)		0.48	0.11	0.24	0.01	0.20	0.09	
Mean (mg/kg)		2.58	0.41	0.51	1.05	0.52	0.47	28.59
proportion (%)	Pi≤1	29.41	97.65	93.98	63.79	96.39	94.05	
	1< Pi ≤2	41.18	2.35	6.02	18.97	3.61	3.57	
	2< Pi ≤3	10.59	0	0	8.62	0	2.38	
	3< Pi ≤5	8.24	0	0	6.9	0	0	
	Pi>5	10.59	0	0	1.72	0	0	
Pollution rate (%)		70.59	2.35	6.02	36.21	3.61	5.95	

**Table 4:** Evaluation of geo-accumulation index.

Element	Min (mg/kg)	Max (mg/kg)	Mean (mg/kg)	Proportion of Pollution Level (%)							Contamination Rate (%)
				I < 0	0 ≤ I < 1	1 ≤ I < 2	2 ≤ I < 3	3 ≤ I < 4	4 ≤ I < 5	>5	
Mn	−2.75	1.71	−1.04	85.9	10.6	3.5	0.0	0.0	0.0	0.0	14.1
Co	−2.13	0.28	−0.78	98.8	1.2	0.0	0.0	0.0	0.0	0.0	1.2
Cu	−1.36	4.60	−0.31	77.6	20.0	1.2	0.0	0.0	1.2	0.0	22.4
Zn	−1.57	3.57	−0.35	80.0	18.8	0.0	0.0	1.2	0.0	0.0	20.0
As	−2.59	2.13	−0.72	85.7	9.5	2.4	2.4	0.0	0.0	0.0	14.3
Cd	−0.50	6.96	1.97	2.4	15.3	42.4	22.4	10.6	3.5	3.5	97.6
Pb	−0.75	2.70	0.02	64.2	27.2	7.4	1.2	0.0	0.0	0.0	35.8
Hg	−4.96	7.60	0.81	39.0	13.6	18.6	15.3	10.2	1.7	1.7	61.0

**Table 5:** Correlation coefficients between PTEs in soils.

Element	Mn	Co	Cu	Zn	Cd	Pb	As	Hg
Mn	1	0.625 **	0.446 **	0.431 **	0.428 **	0.010	0.362 **	0.084
Co		1	0.425 **	0.433 **	0.409 **	−0.042	0.278 *	0.112
Cu			1	0.973 **	0.934 **	−0.126	0.601 **	0.128
Zn				1	0.953 **	−0.031	0.606 **	0.110
Cd					1	0.032	0.626 **	0.095
Pb						1	0.207	−0.033
As							1	0.041
Hg								1.000

* Correlation is significant at the 0.05 level, ** Correlation is significant at the 0.01 level.

**Table 6:** Rotated component matrix for PTEs in soils.

Elements	Component			
Zn	0.946	0.231		
Cd	0.942	0.216		
Cu	0.940	0.236	−0.146	
As	0.721	0.167	0.363	
Mn	0.262	0.874		
Co	0.224	0.860		
Pb			0.970	
Hg				0.996
Eigenvalues	3.309	1.691	1.102	1.005
Percentage of variance (%)	41.362	21.137	13.779	12.564
%Var.acum.	41.362	62.499	76.278	88.841
